# Social Cognition in Autism and Other Neurodevelopmental Disorders: A Co-twin Control Study

**DOI:** 10.1007/s10803-019-04001-4

**Published:** 2019-04-10

**Authors:** J. Isaksson, A. Van’t Westeinde, É. Cauvet, R. Kuja-Halkola, K. Lundin, J. Neufeld, C. Willfors, S. Bölte

**Affiliations:** 1 Center of Neurodevelopmental Disorders (KIND), Division of Neuropsychiatry, Department of Women’s and Children’s Health, Center for Psychiatry Research, Karolinska Institutet & Region Stockholm, Stockholm, Sweden; 2 Child and Adolescent Psychiatry, Region Stockholm, Stockholm, Sweden; 3 Department of Neuroscience, Child and Adolescent Psychiatry Unit, Uppsala University, Uppsala, Sweden; 4 Department of Medical Epidemiology and Biostatistics, Karolinska Institutet, Stockholm, Sweden

**Keywords:** Autism spectrum disorder, Twins, Movie for the assessment of social cognition, ADHD, RATSS

## Abstract

Alterations in social cognition (SC) are hypothesized to underlie social communication and interaction challenges in autism spectrum disorder (ASD). The aetiological underpinnings driving this association remain unclear. We examined SC in 196 twins with ASD, other neurodevelopmental disorders or typical development using the naturalistic Movie for the Assessment of Social Cognition. Autism and its severity were assessed with the Autism Diagnostic Observation Schedule-2, and autistic traits with the Social Responsiveness Scale-2. Using within twin-pair regression models, controlling for age, sex, IQ, and unmeasured familial confounders such as genetic background and shared-environment, SC correlated with ASD diagnosis, autism severity, and autistic traits. Our findings highlight the importance of SC alterations in autism and suggest a non-shared environmental impact on the association.

**Electronic supplementary material:**

The online version of this article (10.1007/s10803-019-04001-4) contains supplementary material, which is available to authorized users.

## Introduction

Autism Spectrum Disorder (ASD) is a common neurodevelopmental condition with an estimated prevalence of 1–2.5% among children, adolescents and young adults (Christensen et al. 2016; Idring et al. 2015). ASD is characterized by the presence of functionally disabling restricted, repetitive behaviours and interests as well as social communication and interaction challenges (Hirvikoski et al. 2016; Järbrink 2007; Knapp et al. 2009). Various models of altered cognitive processing underlying domains of the ASD phenotype have been hypothesized. For instance, alterations of executive control and low levels of endogenous noise in neural signalling have been hypothesized to fuel restricted, repetitive behaviors (Pellicano 2012; Davis and Plaisted-Grant 2015); whereas alterations in sensation and perception, with local information processing bias and hypo-experience based cognition, are presumed to underlie autism related talents such as an eye for details (Happé and Frith 2006; Pellicano and Burr 2012). Social communication and interaction difficulties are presumed to be underpinned by alterations in social cognition (SC) (Brunsdon and Happé 2014; Happé et al. 2017), referring to mental processes relevant for the understanding of agents and their interactions including the self. The term encompasses a wide range of cognitive processes, such as social motivation, emotion recognition, social attention and social learning (Happé et al. 2017). It also includes the ability to attribute mental states and intentions to oneself and others, an ability often referred to as cognitive empathy, mentalization or theory of mind (Sodian and Thoermer 2008; Happé et al. 2017). In typical development, an implicit processing of social information is present at an early age. Implicit SC is characterised as an unconscious, heuristic based and automatic process without deliberate reflection. Later in life, with cognitive and linguistic development, an explicit form of SC, based on deliberate, verbal, rational and conscious consideration of mental states takes form (Heyes and Frith 2014; Happé et al. 2017).

In order to test SC with sufficient sensitivity to detect impairments in intellectually able individuals with ASD, tasks measuring subtle and naturalistic social constellations are needed (Brundson and Happé 2014, Schaller and Rauh 2017). Even though ASD has been reliably associated with alterations in SC, the etiological pathways constituting the relation remain unclear. Twin designs are informative to investigate the relative contributions of genes and environment on SC and autism phenotypes. A population-based twin study of 5-year-olds found that shared and non-shared environmental factors explained most of the variation (93%) in SC scores with genetic influences accounting for only 7% (Hughes et al. 2005). The study used explicit tasks in order to test SC, tapping the child’s ability to attribute mistaken belief to a story character about an object’s identity or location, to predict an action based on attributed false belief and how the character would feel based on his/her false belief. Similar results were reported by another twin study (Ronald et al. 2006), where environmental influences, foremost non-shared, accounted for most of the variation in 9-year-olds attribution to the characters’ thoughts and feelings on the Strange Stories test. In this classic SC test the participant is presented with vignettes of social interactions and asked to explain why a character says something that is not literally true, thus testing the ability to infer mental states in the character. In both studies, SC performance was closely associated with verbal ability. Girls outperformed boys in the study by Hughes et al. (2005), but not in Ronald et al. (2006).

In conclusion, while SC alterations in ASD, and environmental contributions to SC in population-based twin studies are well established, the genetic and environmental influences to SC in relation to clinical ASD and its severity as well as the expression of autistic traits remain unclear. Moreover, other neurodevelopmental disorders (NDDs), such as Attention-Deficit/Hyperactivity Disorder (ADHD), have also been associated with social interaction (Nijmeijer et al. 2007) and SC challenges, as measured with the Reading Mind in the Eyes Test (Baribeau et al. 2015). Thus, further investigation is required on which alterations in SC, and operationalisations of SC, differentiate between ASD and other NDDs. In this study, we test if alterations in SC predict clinical ASD, autism severity and autistic traits for the first time in a clinically enriched twin sample, while adjusting for factors shared between twins in a pair, genetics and family environment. In monozygotic (MZ) twin-pairs the adjustment for genetic factors is maximal, since they are genetically identical. Thus, remaining associations in MZ-twin pairs are not attributable to genetic and shared environmental factors, but to factors unique to an individual. We hypothesized (i) group differences in SC, with lower SC scores in the ASD group compared to typically developing individuals (TD), and individuals with ADHD and other NDDs; (ii) a negative association between SC and clinical ASD diagnosis, autism severity and autistic traits, both across and within-pairs. Additionally, (iii) we explore the contribution of genetic and non-shared environmental factors to the association between SC and ASD diagnosis, autism severity and autistic traits by restricting the sample to MZ twin-pairs only.

## Methods and Materials

### Participants

Within the *Roots of Autism and ADHD Twin Study Sweden* (RATSS), described elsewhere in detail (Bölte et al. 2014b), twin pairs where one or both twins have been screened positively for ASD or ADHD (e.g. the Child and Adolescent Twin Study Sweden [CATSS], Anckarsäter et al. 2011), as well as TD twins, are comprehensively clinically phenotyped. Zygosity is determined on a panel of 48 single nucleotide polymorphisms (Hannelius et al. 2007). Sample characteristics and demographics are summarized in Table 1. In this study, all twins from RATSS of same-sex, above 11-years of age and assessed with the Double Movie of the Assessment of Social Cognition—Multiple Choice (MASC; Bölte et al. 2014a) were included. In total, N = 196 [98 twin pairs; 61 MZ, 37 dizygotic (DZ)] were included (52% females; mean age = 19.39, SD = 4.77, range 12–31). Of these, 40 had primary ASD (20 females, 20 males) of which 15 also had ADHD, 19 primary ADHD (7 females, 12 males), 11 had other NDDs (e.g. communication disorders, specific learning disorders or motor disorders) as their primary diagnosis (4 females, 7 males), and 122 had no NDDs and were categorized as TD in the study (71 females, 51 males). Further, four had a diagnosis of intellectual disability and no other NDD (all male).

**Table 1:** Twin sample characteristics and study variables

	Total (*n* = 196)	Male (*n* = 94)	Female (*n* = 102)
Years of age mean (*SD*), range	19.4 (4.8), 12–31	18.5 (4.4), 12–31	20.2 (5.0), 12–29
IQ Mean (*SD*), range	97.1 (16.4), 62–142	97.1 (15.6), 62–131	97.1 (17.2), 63–142
Autism symptom severity^a^ mean (*SD*), range	2.5 (2.3), 1–10	2.9 (2.5), 1–10	2.1 (2.1), 1–9
Autistic traits^b^ mean (*SD*), range	37.0 (31.5), 0–132	38.9 (32.0), 1–132	35.3 (31.2), 0–128
Social cognition^c^ mean (*SD*), range	30.0 (6.4), 7–41	28.9 (7.0), 10–41	31.0 (5.8), 7–41
Primary ASD diagnosis *N*	40	20	20
Primary ADHD diagnosis *N*	19	12	7
Other pirmary NDD diagnosis *N*	11	7	4
Intellectual disability N	4	4	0
Zygosity MZ/DZ *N*	122/74	52/42	70/32
ASD diagnosis/zygosity (MZ/DZ) *N*	23/17	8/12	15/5
ASD discordant pairs *N*	38	20	18
ASD concordant pairs *N*	16	6	10
ASD discordant pairs/zygosity (MZ/DZ) *N*	18/20	10/10	8/10
ASD concordant pairs/zygosity (MZ/DZ) *N*	12/4	2/4	10/0

*DZ* dizygotic, *MZ* monozygotic

^a^Measured with the Autism Diagnostic Observation Schedule-2 comparison scores

^b^Measured with parental reports on the Social Responsiveness Scale-2

^c^Measured with the Movie of the Assessment of Social Cognition

### Diagnostic and Behavioural Assessments

A clinical consensus diagnosis of ASD (according to DSM-5) was supported by results from medical history and by first choice standardized diagnostic tools, such as the Autism Diagnostic Interview—Revised (ADI-R) (Lord et al. 1994; Zander et al. 2017) and the Autism Diagnostic Observation Schedule Second Edition (ADOS-2, modules 3 & 4) (Gotham et al. 2007; Hus and Lord 2014; Zander et al. 2016). Autism symptom severity was operationalized using ADOS-2 comparison scores, ranging from 1-10, with higher scores indicating more severity. The total score (max. 195) of the parent-report version (standard and adult) of the Social Responsiveness Scale Second Edition (SRS-2) (Constantino and Gruber 2012) was applied to measure autistic traits, with higher total raw scores indicating more autistic traits. Results from adult and child versions of SRS-2 were merged, since the items are compatible and the Kolmogorov-Smirnov test revealed that the distributions were not significantly different (*p* = 0.18).

#### Other Neurodevelopmental Disorders and Intellectual Ability

Clinical DSM-5 consensus diagnosis of ADHD, and other NDDs were determined by a group of clinicians during a 2½ day visit at a clinical research unit using a multitude of documentation including results from the Kiddie Schedule for Affective Disorders and Schizophrenia (Kaufman et al. 1997), the Diagnostic Interview for ADHD in adults (Kooij 2010) or the Structured Clinical Interview for DSM-IV (SCID, axis I), depending on the participant’s age. A diagnosis of intellectual disability was endorsed by results from the Wechsler Intelligence Scales for Children or Adults-IV (WISC-IV/WAIS-IV), the Leiter-revised scales in combination with the Peabody Picture Vocabulary Test Third Edition (in cases of low verbal abilities), as well as the Adaptive Behavior Assessment System-2 (ABAS-2). In addition, a full-scale IQ based on the general ability index on the Wechsler scales was used as a continuous variable.

#### Social Cognition

SC was assessed with the Swedish version of the MASC (Dziobek et al. 2006; Bölte et al. 2014a), a mindreading assessment based on a narrative fictional film with naturalistic verbal and non-verbal stimuli of a dynamic social interaction. The test consists of a 15-min movie of two females and two males meeting on a Saturday night and having dinner together. On the MASC, participants (≥ 12 years of age) are instructed to carefully observe the movie. For testing SC, the film is paused at 43 time-points in critical situations at which questions are asked regarding the film characters’ mental states, emotions, perspectives, and intentions. The test takes approximately 45 min to complete and has been designed to minimize demands on executive functions and central coherence. Four possible answer options are provided for each item, of which only one is a balanced and expected attribution of mental states. Typically expected answers are summed to a total SC mentalizing score, ranging from 0 to 44 with a higher total score indicating increasing SC ability. The MASC also generates three SC subscale scores reflecting tendencies for either excessive mental state attribution (hypermentalizing), reduced SC (hypomentalizing) or a preference for non-social cognition in social context (concrete cognition). Higher scores on the hypermentalizing, hypomentalizing and concrete cognition subscales indicate greater SC challenges. The MASC has recurrently demonstrated sensitivity of SC alterations in adolescents and adults with ASD, compared to TD and other control groups (Dziobek et al. 2006; Müller et al. 2016; Lahera et al. 2014; Schaller and Rauh 2017; Martinez et al. 2017). It has also been shown to have high internal consistency, interrater and retest reliability as well as discriminant validity (Dziobek et al. 2006; Bölte et al. 2014a).

### Design and Statistical Analyses

**Fig. 1:**
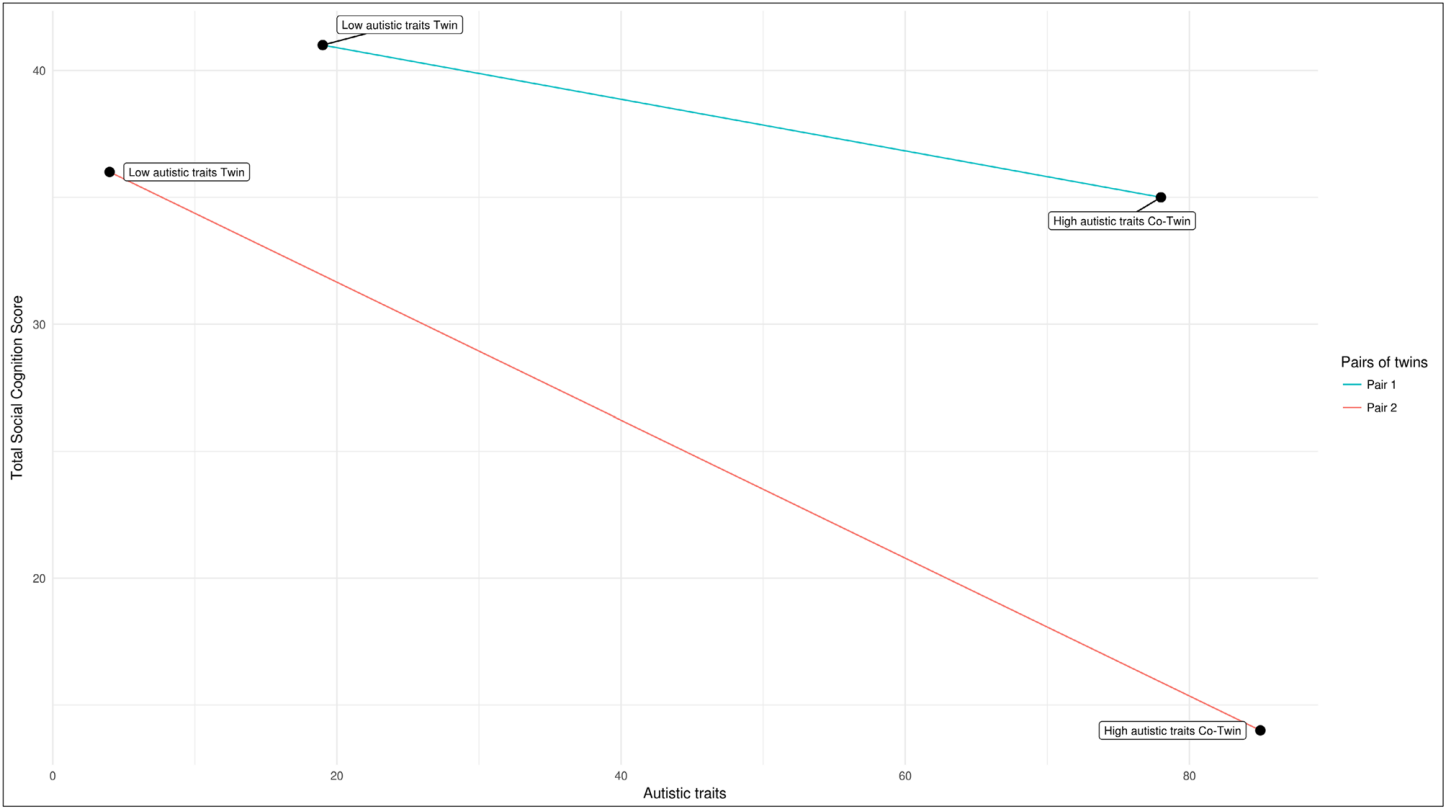
Within-pair association between autistic traits and social cognition in two example pairs

**Fig. 2:**
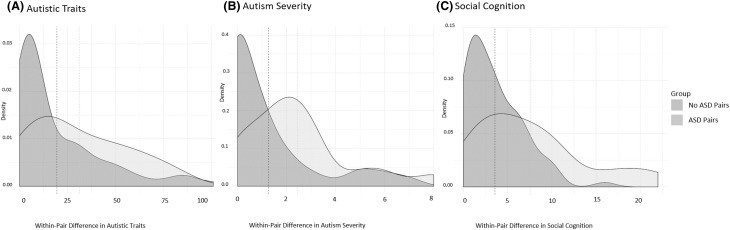
Within-pair difference in the distributions on autistic traits, autism severity and social cognition

To compare participants with a primary ASD diagnosis, primary ADHD (without ASD), other primary NDD diagnosis (without ASD/ADHD) or TD, as well as males and females, on MASC performance (total mentalizing score, hyper-and hypomentalizing, concrete cognition), we used Kruskal–Wallis tests since the data showed a skewed distribution (Shapiro–Wilk test of normality*: W* = 0.941, *p* < 0.001). As a post-hoc test we compared the different groups separately using the Mann-Whitney test. Next, associations between SC and ASD, autism severity and autistic traits were analyzed using linear (continuous outcomes) and logistic (binary outcome) regressions in a generalized estimating equations (GEE) framework that has been developed to fully account for twin/co-twin designs and allowing both categorical and continuous data (Neuhaus and McCulloch 2006), using the drgee package (v. 1.1.5) in R (v. 3.3.1). We estimated associations between SC and autism outcomes adjusting for age, sex and IQ. However, because of the non-random sampling scheme for the current sample, it is uncertain whether estimates from these standard analyses generalize to a normal, non-selected, population. Fortunately, the sample is appropriate to analyse in so-called within-analyses using conditional regressions (conditional linear and conditional logistic regressions). Thus, within-pair analyses (twin/co-twin analysis) were performed; in these analyses each pair is considered a separate stratum, and analyses may be interpreted as within-pair differences in outcomes (ASD-related variables) being regressed on within-pair differences in exposure (SC) and covariates (see Fig. 1). Importantly, the within-pair analyses adjust for all shared factors within the pair, including unmeasured, thus familial confounding factors (shared environment and genetic) are adjusted for (as well as shared mediators, for a detailed investigation see Sjölander and Zetterqvist 2017). The within-pair distributions of SC performance, autism severity and autistic traits are displayed in Fig. 2. Finally, we re-calculated the within-pair analyses for MZ twin-pairs only, in order to investigate the robustness of results when genetic confounding was completely adjusted. Two tailed tests with *p* values < 0.05 were considered significant. If applying Bonferroni correction, based on the number of main analyses, the null hypothesis is rejected if the *p* < 0.02.

## Results

### Group Differences in Social Cognition

**Fig. 3:**
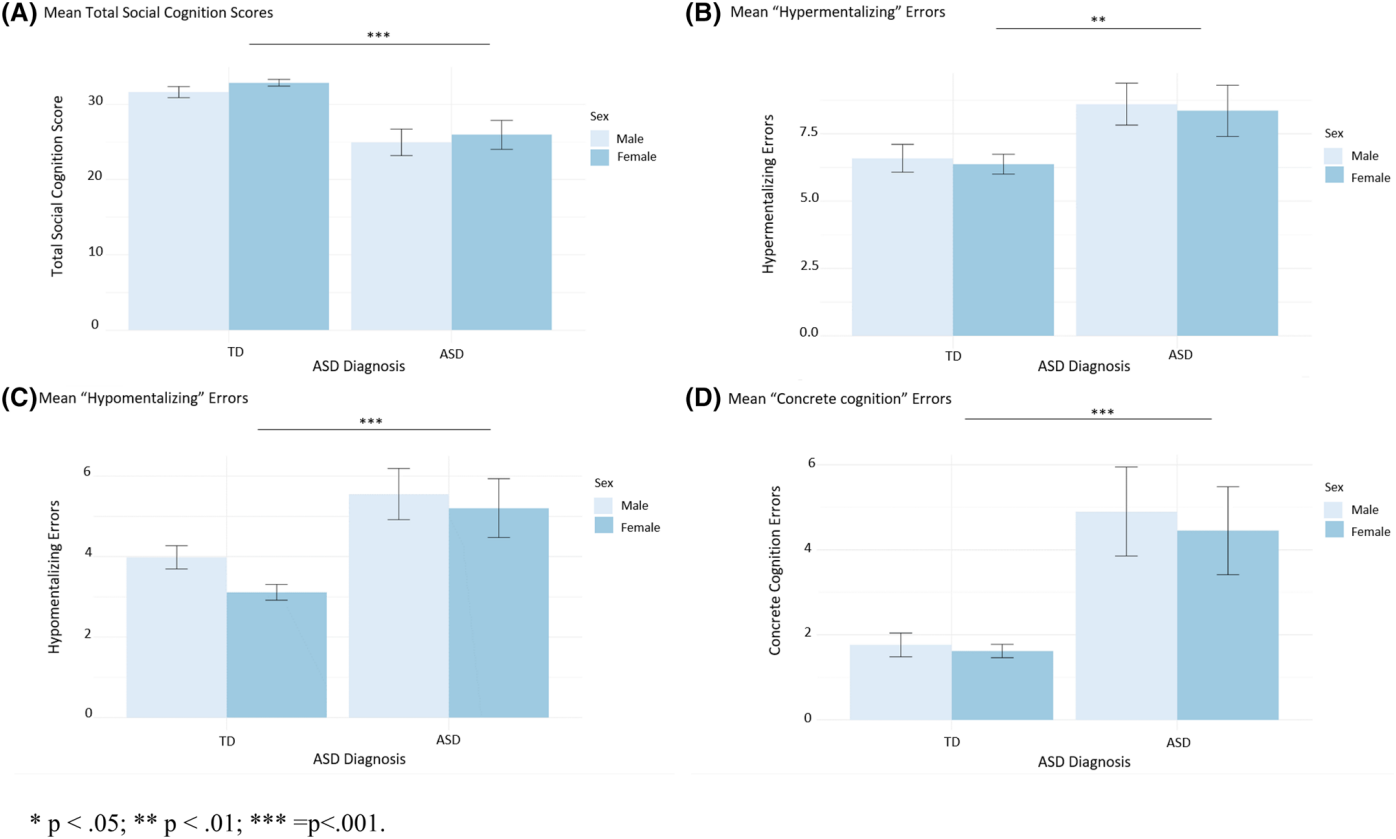
Differences between ASD cases and typically developing individuals (TD) on social cognition scores. *p < 0.05; **p < 0.01; ***p<0.001

The ASD, ADHD, other NDD and TD groups differed for SC ability (*χ*^*2*^(4) = 39.17; *p* < 0.001), hypermentalizing (*χ*^*2*^(4) = 16.31, *p* = 0.003), hypomentalizing (*χ*^*2*^(4) = 24.35; *p* < 0.001) and concrete cognition (*χ*^*2*^(4) = 27.94; *p* < 0.001), with ASD cases exhibiting a reduced total SC ability (*p* < 0.001), more hypermentalizing (*p* = 0.002), hypomentalizing (*p* < 0.001) and concrete cognition (*p* < 0.001) compared to TDs (Table 2 and Fig. 3). These results remained significant when excluding participants with ID. Only the ASD cases, as opposed to participants with ADHD and other NDDs, showed more hypomentalizing and concrete cognition compared to TDs (Table 2). There were no differences in SC scores between the ASD group, ADHD group and other NDDs. Females had higher SC abilities than males (*p* = 0.033) and showed less hypomentalizing (*p* = 0.003). More specifically, these sex differences were driven by TDs (for SC ability, *p* = 0.037; hypomentalizing, *p* = 0.003), and were not observable among ASD participants.

**Table 2:** Group and post-hoc comparison for primary diagnosis on social cognition scores (Mean and SD) as measured with the Movie of the Assessment of Social Cognition

Social cognition per diagnosis	ASD (N = 40)	ADHD (N = 19)	Other NDD (N = 11)	TD (N = 122)	Kruskal–Wallis group-comparison	Wilcoxin post-hoc comparison
Total social cognition score	25.45 (8.16)	28.53 (4.56)	27.55 (6.64)	32.34 (4.52)	*Χ*^*2*^(4) = 39.17, *p* < 0.001	ASD < TD, *p* < 0.001 ADHD < TD, *p* < 0.001 Other NDD < TD, *p* = 0.013
Hypermentalizing	8.48 (3.83)	8.63 (3.55)	8.64 (4.37)	6.46 (3.34)	*χ*^*2*^(4) =16.31, *p* = 0.003	ASD > TD, *p* = 0.002 ADHD > TD, *p* = 0.013
Hypomentalizing	5.38 (3.03)	4.32 (1.89)	4.73 (2.37)	3.48 (1.88)	*χ*^*2*^(4) = 24.35; *p* < 0.001	ASD > TD, *p* < 0.001
Concrete cognition	4.68 (4.59)	2.53 (2.46)	3.09 (2.47)	1.68 (1.63)	*χ*^*2*^(4) = 27.94; *p* < 0.001	ASD > TD, *p* < 0.001

### Association Between Social Cognition and Autism

**Fig. 4:**
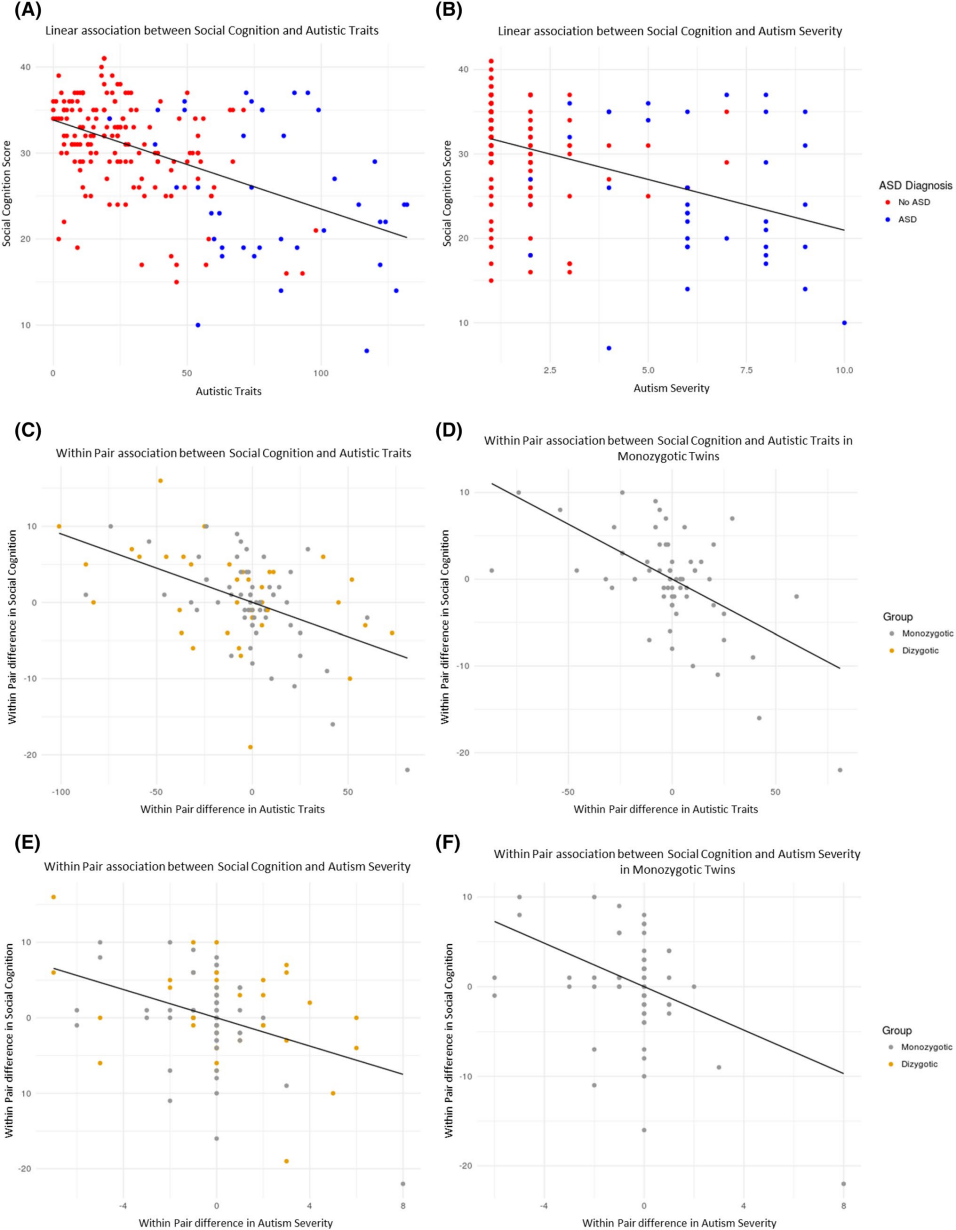
Association between social cognition, autistic traits and autism severity

Alterations in SC ability were associated with an increase in autism symptom severity (*β* = − 0.14, *p* < 0.001, *CI* − 0.20, − 0.07) and autistic traits (*β* = − 2.04, *p* < 0.001, 95% *CI* − 2.90, − 1.18) (Fig. 4 & Supplementary Table 1). There were no statistically significant main effects of IQ, age or sex, and no interaction between sex and SC, on autism symptom severity or autistic traits. In the logistic regression, with ASD diagnosis as outcome, the results remained similar for SC (*β* = − 0.14, *p* < 0.001, *CI* − 0.22, − 0.06). Concrete cognition was associated with higher autism severity (*β* = 0.23, *p* < 0.001, *CI* 0.10, 0.36), an increase of autistic traits (*β* = 3.20, *p* < 0.001, *CI* 1.61, 4.78) and ASD diagnosis (*β* = 0.23, *p* < 0.001, *CI* 0.10, 0.38). In addition, hypomentalizing was associated with autism severity (*β* = 0.28, *p* < 0.001, *CI* 0.13, 0.42) and autistic traits (*β* = 2.53, *p* = 0.041, *CI* 0.10, 4.97). Further, hypermentalizing was associated with autistic traits (*β* = 1.33, *p* = 0.050, *CI* 0.00, 2.66).

### Association Between Social Cognition and Autism Within-Pairs

A within-pair reduction of SC ability was associated with a within-pair increase in autism severity (*β* = − 0.13, *p* = 0.009, *CI* − 0.22, − 0.03), autistic traits (*β* = − 2.09, *p* < 0.001, *CI* − 3.10, − 1.08) and ASD diagnosis (*β* = − 0.22, *p* = 0.035, *CI* − 0.42, − 0.02) (Fig. 4 & Supplementary Table 1). Hypermentalizing was associated with autistic traits (*β* = 2.21, *p* < 0.001, *CI* 1.13, 3.30) and ASD diagnosis (*β* = 0.29, *p* = 0.031, *CI* 0.026, 0.55), while hypomentalizing was associated with autism severity (*β* = 0.27, *p* = 0.002, *CI* 0.10, 0.45).

### Association Between Social Cognition and Autism Within MZ-Pairs Only

Adjusting for IQ, a within-pair decrease in SC ability was associated with a within-pair increase in autism severity (*β* = − 0.11, *p* = 0.021, *CI* − 0.21, − 0.18) and autistic traits (*β* = − 2.08, *p* < 0.001, *CI* = 2.95, = 1.20) in monozygotic twins, as well as a trend for ASD diagnosis (*β* = − 0.28, *p* = 0.058, *CI* − 0.56, 0.01). In addition, more hypermentalizing (*β* = 1.97, *p* = 0.013, *CI* 0.41, 3.52), hypomentalizing (*β* = 2.75, *p* = 0.031, *CI* 0.25, 5.24) and concrete mentalizing (*β* = 2.39, *p* = 0.023, *CI* 0.34, 4.45) were associated with an increase in autistic traits in MZ pairs.

## Discussion

This study is the first to use a co-twin control approach to examine the relationship between SC, on one hand, and ASD, autism symptom severity and autistic traits, on the other. We used a naturalistic social cognition assessment tool, and found, as expected, ASD cases to have altered SC compared to typically developing participants. Especially, concrete thinking in social context distinguished ASD from TD, compared to participants with ADHD or other NDDs. In the TD group, but not in the ASD group, females outperformed males on SC. Consistent with our hypotheses, a robust association between reduced SC, ASD, autism symptom severity and autistic traits, both between and within the pairs was found. This association between alterations in SC and autism phenotypes was independent of sex and IQ, and largely remained in the MZ twins.

Our findings are in line with previous reports of an association between ASD and challenges in SC in general (Brunsdon and Happé 2014), as well as studies where SC is operationalized by the MASC specifically (Dziobek et al. 2006; Lahera et al. 2014; Schaller and Rauh 2017). The MASC has shown to discriminate between adolescents/adults with Asperger syndrome/ASD and TD volunteers, both for overall mentalizing as well as on all its subscales. Further, we show that alterations in SC are associated not only with an ASD diagnosis, but also autism severity and autistic traits in a linear model across the sample. Occasional negative findings on SC alterations in ASD (Brunsdon and Happé 2014; Pellicano et al. 2006), might to some extent reflect the wide variety of tasks used to measure SC, including tasks of limited sensitivity of subtle SC alterations. The MASC has shown to be superior to other established SC tools in detecting SC alterations in ASD (Schaller and Rauh 2017), possibly since the task captures a more complex framework of social interactions. In our study, participants with ADHD and other NDDs also scored lower on MASC compared to TDs, a result that is in line with a recently published meta-analysis where ADHD cases preformed at an intermediate level between ASD and TD (Bora and Pantelis 2016). Only the ASD group, as compared to ADHD and other NDDs, differed from TDs on having more hypomentalizing and concrete mentalizing.

Thus far, there have been few studies on the fundamental determinants of SC variation. Here, we used both a between- and a within-pair twin design to analyse the association between SC, ASD, autism symptom severity and autistic traits in terms of genetic, shared- and non-shared environmental contributions. As hypothesized, reduced SC performance was associated with ASD diagnosis, autism symptom severity and autistic traits in the between-pair model, as well as in the within-pair model. Thus, even after maximal control for shared factors, such as sex, age, socioeconomic status and shared family environment in the within-pair model, the association between SC and autism remained, highlighting the robust nature of the association of SC and autism from a clinical and continuous conception viewpoint. Moreover, when only including MZ twins, controlling for genetic background, the relationship between SC, autism symptom severity and autistic traits remained. Thus, in line with Hughes et al. (2005) and Ronald et al. (2006), who found a non-shared environment effect on SC our results indicate a non-shared environment impact on the SC/autism association. In addition, autism was associated with higher scores on all of the MASC subscales, suggesting that SC challenges are not only restricted regarding mental state attribution (concrete or hypomentalization), but also to excessive mental state attribution (hypermentalization), a pattern that has also been found in other conditions, such as schizophrenia (Bliksted et al. 2018), and are consistent with previous SC research using the MASC (Lahera et al. 2014; Martinez et al. 2017). It may be concluded that autism is linked to SC insecurity, leading to both over- und underestimations of social context, rather than a widely assumed social context neglect.

The association between reduced SC ability and autism, within the MZ pairs, was limited to autism symptom severity and autistic traits, with only a trend for categorical ASD. Thus, our data supports the notion that quantitative approaches to autism might more adequately describe autism phenotypes and also result in more informative or sensitive research findings (Robinson et al. 2016; Ronald et al. 2006). Finally, the association between SC, ASD diagnosis, autism symptom severity and autistic traits remained after adjusting for sex as well as IQ. We did, however, observe sex differences on mean SC performance, with males showing more reduced mentalizing capacities, particularly hypomentalizing, which is in line with the Empathizing-Systemizing theory, where autism reflects an “extreme-male” form of cognition (Baron-Cohen 2002). In addition, both being male as well as lower IQ have previously been associated with lower MASC scores (Müller et al. 2016) and previous results using the MASC had found that these sex differences in SC might be driven by females being particularly superior to males in judging the SC of women (Wacker et al. 2017). Interestingly, the observed sex differences in reduced mentalizing capacities were limited to participants without any NDDs, whereas males and females with ASD showed no differences in mentalizing capacity. This finding may endorse the validity of the MASC, and confirm its sensitivity for social cognition challenges in autistic girls and women, who have been suggested to be missed by standard diagnostic procedures, owing to social camouflage (Rynkiewicz et al. 2016). However, the fact that we did not find sex-differences in the ASD subsample may also reflect a sample bias. That is, our study might have primarily included female autistic participants showing a low degree of social camouflaging behaviour. Our results suggest that despite general sex differences in SC, reduced SC is associated with ASD, autism symptom severity and autistic traits across sexes, and independent of IQ.

As to limitations, although this is a reasonably sized twin study using deep phenotyping (Bölte et al. 2014b), the study would have benefitted from a larger sample size, adding power to the models. The non-significant finding in the MZ population for SC and ASD as a categorical diagnosis, probably reflects the smaller sample size within this population, with *only* N = 18 discordant pairs. Also, the size of our clinically enriched and carefully phenotyped sample, is too small for common heritability models that require large population-based twin samples, such as the ACE model. In addition, the comparison based on primary diagnosis resulted in small sample sizes, and only *n*= 11 participants in the other NDDs group, which is why Kruskal–Wallis test for the non-normal distributions were used. Recruitment for this study were made based on any possible NDD (or being TD). Thus, our sample is not randomly sampled from the general population and the generalizability of the between-pair analyses (the within-pair analyses is not affected by the skewed sampling) is not necessarily straightforward for non-clinical populations. Concerns have also been raised that findings in twin samples may not be generalizable to non-twin populations. However, an investigation of psychiatric illnesses showed no differences between twins and non-twins (Kendler et al. 1996), and the prevalence of autism diagnoses is similar in twins and non-twin full siblings in Sweden (Sandin et al. 2014). Research comparing SC in twins and to non-twins and their siblings found no significant differences concerning SC performance (Wright Cassidy et al. 2005). The current study used a single measure to operationalize SC, mostly covering explicit SC based on verbal, rational and conscious consideration of mental states. A measure that directly targets more implicit aspects of SC might be even more informative to explore SC in ASD since autistic individuals in the average to high IQ range may be able to acquire explicit SC, but not implicit SC skills, by training and over time (Bölte et al. 2015). However, the MASC is probably not a test purely tapping on explicit SC, as it includes understanding of multiple, subtle, naturalistic social interactions in a contextual framework, also requiring at least some form of implicit social processing, such as the use of schemes and scripts (Schaller and Rauh, 2017). In addition, the subscales allow for further investigation into alterations of SC, such as hypo-, hyper- and concrete mentalization. Moreover, although the MASC has shown to be a sensitive and valid measure of SC, even in comparison to other established mentalizing measures, additional assessment of SC also covering other dimensions of SC, such as social orientation and motivation, are desirable in future research. Lastly, the TD twins in this study were TD in the sense that they did not have any NDDs. However, other psychiatric diagnoses, such as a history of depression or anxiety disorder, were not an exclusion criteria. However, other psychiatric symptoms may also be present in the ASD group, thereby increasing the comparison between the two group. In addition, since psychiatric symptoms such as depression (Bora and Berk, 2016) and PTSD (Plana et al. 2014) have been associated with SC problems, the results may be even more robust compared to having a control group without any mental health problems.

The present study combines several strengths, including the assessment of both autistic traits, which are normally distributed within the population, using the SRS, and clinician rated autism symptoms and their severity using the ADOS, resulting in categorical clinical decisions. By using ratings from both parents and clinicians the risk of rater bias is reduced. The results, using either parental or clinician ratings, were mostly comparable although some differences were found for the subscales, with hypermentalization being mostly related to parental ratings. For future studies, it would be of interest to include possible non-shared environmental factors that may contribute to the association, e.g. birth weight which often varies between the twins and has been shown to predict SC at 4.5 years of age (Wade et al. 2014). Further, future studies should address whether more implicit or explicit forms of SC are associated with autistic traits, and if these aspects of social cognition are differentially influenced by genetic, shared- and non-shared environmental factors.

To conclude, by using co-twin design we show for the first time that alterations in social cognition ability are associated with autism, autism severity and autistic traits, independent of sex, IQ and familial confounders. The finding suggest a non-shared environmental effect underpinning the associations and stresses the importance of SC for autism phenotypes. We found that autism is closely linked with SC, not only reduced mental state attribution, which is a widespread belief, but also to excessive mental state attribution. There are today several training programs targeting SC in ASD populations, such as computer-based facial affect recognition training (Fridenson-Hayo et al. 2017) and group-based social communication or skills training (Choque Olsson et al. 2017). Given the importance of SC for ASD, as well as the non-shared environmental contribution to this association, implementation of SC training in clinical practice could have beneficial effects for children and young adults with ASD.

## Electronic supplementary material

Below is the link to the electronic supplementary material.

Supplementary material 1 (DOCX 20 kb)
