# Supplementary material for: Social Cognition in Autism and Other Neurodevelopmental Disorders: A Co-twin Control Study
Source: J Autism Dev Disord. 2019 Apr 10;49(7):2838–48. doi: 10.1007/s10803-019-04001-4 (PMC6606667; doi:10.1007/s10803-019-04001-4)
Supplement: Supplementary file 1 — Supplementary material 1 (DOCX 20 kb) [file 10803_2019_4001_MOESM1_ESM.docx]

**Supplementary Table 1** Results for the cohort (Between Pair) GEEs and Within Pair GEEs, with scores on the Movie of the Assessment of Social Cognition (total score, hypermentalization, hypomentalization and concrete cognition separately) predicting autistic traits as measured with the Social Responsiveness Scale-2 total score (SRS-2), autism severity as measured with the Autism Diagnostic Observation Schedule-2 comparison scores (ADOS-2) and ASD diagnosis. Between Pair model is adjusted for IQ, Sex and Age, Within Pair model is adjusted for IQ.

| **BETWEEN PAIR MODEL** | | | | | | |
| --- | --- | --- | --- | --- | --- | --- |
|  | **Autism Traits (SRS-2)** | | **Autism Severity (ADOS-2)** | | **ASD diagnosis** | |
|  | ***β (SD)*** | ***95 % CI*** | ***β (SD)*** | ***95 % CI*** | ***β (SD)*** | ***95 % CI*** |
| Social Cognition Score | *-2.04 (0.44)**** | -2.90, -1.18 | *-0.14 (0.03)**** | -0.20, -0.07 | *-0.14 (0.04)**** | -0.22, -0.06 |
| IQ | *-0.24 (0.19)* | -0.61, 0.12 | *-0.004 (0.02)* | -0.03, 0.03 | *-0.005 (0.02)* | -0.05, 0.04 |
| Sex | *1.21 (4.82)* | -8.25, 10.67 | *-0.42 (0.36)* | -1.13, 0.29 | *0.19 (0.46)* | -0.71, 1.08 |
| Age | *-0.26 (0.51)* | -1.25, 0.73 | *-0.01 (0.05)* | -0.10, 0.08 | *0.04 (0.05)* | -0.05, 0.14 |
| Hypermentalization^a^ | 1.33 (0.68)* | 0.00, 2.66 | 0.03 (0.06) | -0.09, 0.14 | 0.06 (0.06) | -0.06, 0.17 |
| Hypomentalization^a^ | 2.53 (1.24)* | 0.10, 4.97 | 0.28 (0.07)*** | 0.13, 0.42 | 0.17 (0.10) | -0.03, 0.37 |
| Concrete cognition^a^ | 3.20 (0.81)*** | 1.61 4.78 | 0.23 (0.07)*** | 0.10, 0.36 | 0.23 (0.07)*** | 0.10 0.38 |
| **WITHIN PAIR MODEL** | | | | | | |
|  | **Autism Traits (SRS)** | | **Autism Severity (ADOS)** | | **ASD diagnosis** | |
| **All twins (n=196)** | ***β (SD)*** | ***95 % CI*** | ***β (SD)*** | ***95 % CI*** | ***β (SD)*** | ***95 % CI*** |
| Social Cognition Score | *-2.09 (0.52)**** | -3.10, -1.08 | *-0.13 (0.05)*** | -0.22, -0.03 | *-0.22 (0.10)** | -0.42, -0.02 |
| IQ | -0.67 (0.28)* | -1.21, -0.13 | -0.04 (0.02) | -0.08, 0.00 | -0.02 (0.03) | -0.08, 0.04 |
| Hypermentalization^b^ | 2.22 (0.55)*** | 1.13, 3.30 | 0.06 (0.08) | -0.10, 0.21 | 0.29 (0.13)* | 0.03, 0.55 |
| Hypomentalization^b^ | 1.90 (1.20) | -0.45, 4.25 | 0.27 (0.09)** | 0.10, 0.45 | 0.21 (0.14) | -0.06, 0.48 |
| Concrete cognition^b^ | 1.88 (1.11) | -0.30 4.06 | 0.13 (0.10) | -0.07 0.32 | 0.23 (0.18) | -0.12 0.59 |
| **Monozygotic Twins (n=122)** |  |  |  |  |  |  |
| MASC total score | *-2.08 (0.45)**** | -2.95, -1.20 | *-0.11 (0.05)** | -0.21, -0.02 | *-0.28 (0.15)* | -0.56 0.01 |
| IQ | -0.73 (0.30)* | -1.32, -0.15 | -0.06 (0.03)* | -0.11, -0.01 | -0.02 (0.05) | -0.12, 0.08 |
| Hypermentalization^b^ | *1.97 (0.79)** | 0.41, 3.52 | *0.11 (0.06)* | -0.02, 0.23 | *0.34 (0.27)* | -0.18, 0.86 |
| Hypomentalization^b^ | 2.75 (1.27)* | 0.25, 5.24 | 0.19 (0.10)* | 0.00, 0.39 | 0.45 (0.31) | -0.15, 1.06 |
| Concrete cognition^b^ | 2.39 (1.05)* | 0.34, 4.45 | 0.11 (0.12) | -0.12, 0.34 | 0.34 (0.29) | -0.23, 0.91 |

Note. ^a^ Separate models for subscores on Social Cognition, also adjusted for IQ, sex and age. ^b^ Separate models for subscores on Social Cognition, adjusted for IQ.

* p < .05; ** p < .01; *** =p<.001.
